# Cutaneous and Mucosal Lichen Planus: A Comprehensive Review of Clinical Subtypes, Risk Factors, Diagnosis, and Prognosis

**DOI:** 10.1155/2014/742826

**Published:** 2014-01-30

**Authors:** Farzam Gorouhi, Parastoo Davari, Nasim Fazel

**Affiliations:** Department of Dermatology, University of California Davis, 3301 C Street, Sacramento, CA 95816, USA

## Abstract

Lichen planus (LP) is a chronic inflammatory disorder that most often affects middle-aged adults. LP can involve the skin or mucous membranes including the oral, vulvovaginal, esophageal, laryngeal, and conjunctival mucosa. It has different variants based on the morphology of the lesions and the site of involvement. The literature suggests that certain presentations of the disease such as esophageal or ophthalmological involvement are underdiagnosed. The burden of the disease is higher in some variants including hypertrophic LP and erosive oral LP, which may have a more chronic pattern. LP can significantly affect the quality of life of patients as well. Drugs or contact allergens can cause lichenoid reactions as the main differential diagnosis of LP. LP is a T-cell mediated immunologic disease but the responsible antigen remains unidentified. In this paper, we review the history, epidemiology, and clinical subtypes of LP. We also review the histopathologic aspects of the disease, differential diagnoses, immunopathogenesis, and the clinical and genetic correlations.

## 1. Introduction

Lichen planus (LP) is a chronic inflammatory and immune mediated disease that affects the skin, nails, hair, and mucous membranes. Cutaneous lichen planus (CLP) most commonly involves the flexor surfaces of the extremities and presents as small itchy violaceous Papules in middle-aged adults. “Pruritic, Purple, Polygonal, Planar, Papules, and Plaques” are the traditional 6 “P's” of LP [1]. The lesions are typically bilateral and relatively symmetric. Oral LP (OLP) can be the sole clinical presentation of the disease or accompanied by cutaneous or other mucosal manifestations including the genital area, gastrointestinal tract, and eyes.

## 2. Materials and Methods

In this paper, we review the different aspects of LP, including history, epidemiology, clinical subtypes, histopathologic features, differential diagnoses, immunopathogenesis, clinical and genetic correlations, quality of life, and prognosis of the disease. We searched the literature using terms: “lichen” and “planus.”

## 3. Results and Discussion

### 3.1. History

LP (Greek “Leichen” = tree moss, Latin “planus” = flat, even) [2] was first explained in 1869 by Dr. Wilson as an inflammatory disorder of the stratified squamous epithelia with an unknown etiology. Dr. Wilson probably referred to the condition that was originally described by Herba as “Lichen ruber” [3, 4]. It was originally named “lichen ruber planus” and “lichen psoriasis” [5]. Weyl initially described the characteristic surface markings on LP Papules, known as Wickham striae, in 1885 [6], and Wickham explained it further in 1895 [7]. Darier correlated the presence of Wickham's striae with an increase in thickness of the granular cell layer [8]. In 1937, Guogerot and Burnier described the coexistence of oral, cervical, and stomach LP lesions with no cutaneous involvement as “plurimucosal LP” [9]. In 1982, Pelisse and colleagues reintroduced a similar variant of mucosal LP as the vulvovaginal-gingival syndrome with erosive lesions involving the oral and vulvovaginal mucosa [10].

### 3.2. Epidemiology

The exact prevalence of LP is unknown. Nevertheless, the estimated prevalence of LP is in the range of 0.22% to 5% worldwide [11–15]. The epidemiological studies lack clear diagnostic criteria or a uniform methodology. Furthermore, the diverse clinical presentation and the asymptomatic nature of the most common subtype of OLP make the disease an underdiagnosed health issue [16]. McCartan and Healy [17] identified forty-five studies that calculated the prevalence or incidence of LP. They concluded an overall age-adjusted prevalence of 1.27% (0.96% in men and 1.57% in women) in Sweden [14, 18]. The incidence of LP was 0.032%–0.037% in a British population [19]. LP typically affects middle-aged adults of both genders. No sexual predilection is evident but some reports indicate a slight predominance in women up to a ratio of 2 : 1 [20]. Interestingly, in the top 3 largest case series of childhood LP, the female to male ratio is reported to be 1 : 2 in a US population [21], 1 : 1.5 in an Indian cohort [22], and 2 : 1 in a Canadian study [23]. Such variability may be explained by different inclusion and exclusion criteria within the studies. The relative male predilection in childhood LP is unusual for an autoimmune disease and suggests that other possible unknown mechanisms may be involved in the pathogenesis of LP. Childhood LP is more common in the African American population [23]. Moreover, hypertrophic and actinic variants as well as LP pigmentosus are more prevalent amongst African Americans or darker skinned individuals [2, 24, 25]. Postinflammatory hyperpigmentation is a characteristic outcome of LP lesions that is predominantly more common in the African American population [2]. Asians acquire the follicular form less frequently than the other ethnicities according to a Canadian epidemiological study [26].

### 3.3. Clinical Subtypes

#### 3.3.1. CLP

CLP has different clinical subtypes based on the morphology of the lesions and the site of involvement.

*Subtypes Based on Configuration or Morphology of the Lesions. *They include papular (classic), hypertrophic, vesiculobullous, actinic, annular, atrophic, linear, follicular, LP pigmentosus and LP pigmentosus-inversus.

**Figure 1:**
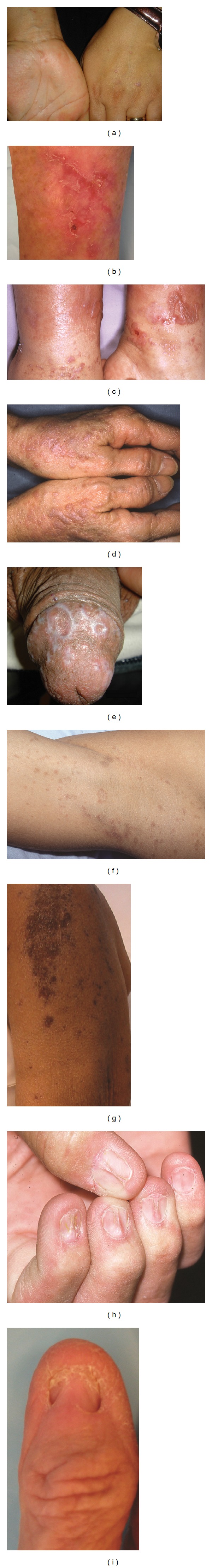
(a) Classic CLP: violaceous Papules on the dorsal hand and volar wrist (courtesy of Dr. Omid Zargari); (b) hypertrophic CLP: centrally eroded hyperkeratotic plaques involving the lower leg; (c) vesiculobullous CLP: vesicles and bullae on right and left ankles and lower legs (courtesy of Dr. Peter Lynch); (d) actinic CLP: hyperpigmented Papules and plaques on the dorsal hands (courtesy of Dr. Peter Lynch); (e) annular CLP: reticulated white striae involving the glans penis (courtesy of Dr. Omid Zargari); (f) atrophic CLP: hyperpigmented macules and patches on the arm (courtesy of Dr. Peter Lynch); (g) zosteriform CLP: linearly oriented confluent violaceous Papules on the arm; (h) nail CLP: longitudinal ridging of the fingernails (courtesy of Dr. Peter Lynch); (i) nail CLP: dorsal pterygium of the thumbnail; (a), (b), (e), (g), and (i) are reprinted with permission from [50]. CLP, cutaneous lichen planus.

The classic CLP lesion is a shiny, red/purple-colored, flat-topped papule (Figure 1(a)). Lesions may also have a thin, transparent, and adherent scale. Wickham's striae, which are defined as fine whitish points or lacy lines, may be seen on the surface of well-developed Papules [27].

Hypertrophic LP is characterized by hyperkeratotic thick pruritic red-brown to purple-gray plaques with follicular accentuation [28] that commonly involves the extremities, especially the anterior legs and the interphalangeal joints in a symmetrical distribution (Figure 1(b)) [2]. Welsh et al. described its resemblance to the extrusive forms of igneous rock as a useful sign in distigushing the hypertrophic subtype from other differential diagnoses [28]. Polygonal Papules may be seen surrounding the main lesion.

In the vesiculobullous subtype, blisters develop within the plaques (Figure 1(c)). Lower extremities are the main site of involvement. This pattern of the disease has to be distinguished from LP pemphigoides, a rare coexistence of LP and bullous pemphigoid [29].

Actinic CLP is a rare subtype presenting as nummular patches or plaques with a hypopigmented halo surrounding a hyperpigmented center (Figure 1(d)). This variant is more prevalent in African Americans, Indians, and Middle-Eastern individuals and affects the sun-exposed areas [30, 31].

Annular CLP is an uncommon form that classically involves the male genitalia (glans penis and penile shaft) and also axilla, groin, and extremities (Figure 1(e)) [32]. Although CLP usually presents with pruritus, the annular form is often asymptomatic especially when arising in the genital area [33].

Atrophic CLP is the clinical endpoint of chronic annular or hypertrophic LP with atrophic lesions (Figure 1(f)) [34]. Diagnosis may be difficult unless classic LP is present elsewhere on the body. The anatomical distribution of lesions may be helpful as it mainly affects the same areas that are involved in the annular or hypertrophic variants. Long-term use of potent or superpotent topical corticosteroids may predispose the patient to developing atrophic lesions.

Linearly oriented lesions of CLP can be caused by the Koebner phenomenon, but this pattern is not considered as the true linear form. The true linear form is more extensive and follows the lines of Blashko [35]. In rare circumstances, if linear LP presents in a dermatomal pattern, it is called zosteriform LP (Figure 1(g)) [36]. This rare variant is found either at the site of healed herpes zoster lesions (Wolf isotopic response) or de novo in normal skin. The exact etiology of the zosteriform subtype remains debatable [37]. The isotopic response can also occur in the annular subtype [38].

LP pigmentosus is usually seen in Indians and darker skinned individuals. Lesions are characteristically bilateral and involve sun-exposed areas [24]. Conversely, LP pigmentosus-inversus was reported in whites and lighter-skinned Asians, which typically affects the intertriginous and flexural areas [39, 40].

Classic follicular CLP, fibrosing alopecia in a pattern distribution, frontal fibrosing alopecia (FFA), and Graham Little-Piccardi-Lassueur syndrome [41]. The typical form of LPP is characterized by pinpoint hyperkeratotic Papules often found on the scalp. It frequently affects the vertex but can also involve other parts of the scalp [42]. LPP has a sexual predilection for females and can involve nail and mucosa [43, 44]. LPP can also be induced by hair transplantation or face-lift surgery. The possible triggering factors include the Koebner phenomenon, perisurgery antigen release, or the postoperative immune-inflammatory response [45, 46]. “Fibrosing alopecia in a pattern distribution” is another suggested subtype that has a centroparietal pattern. It lacks the multifocal scarring of classic LPP and can be discerned accordingly [47]. FFA is more commonly seen in postmenopausal women. The pattern of alopecia is analogous to male pattern androgenetic alopecia with frontal dominance and additional features such as scarring and a lichenoid pattern on histopathology [48]. Graham Little-Piccardi-Lassueur syndrome is a rare variant of LPP characterized by the triad of follicular-based spinous Papules on the body, scalp, or both, patchy cicatricial alopecia of the scalp, and noncicatricial alopecia of the axilla and pubic region. It is preferentially more common in females [49].

*Lichen Planus Subtypes Based on the Site of Involvement. *Palmoplantar LP is a rare subtype. The erythematous scaly form is the most common clinical presentation. The lesions are multiple (more than 10 lesions), bilateral, and often symmetrical with prominent associated pruritus. The most common sites of involvement include the malleoli and also the internal plantar arch [51]. Fingertips are often spared. The second most common clinical form presents as hyperkeratotic plaques [51]. Palmoplantar LP can less frequently present as vesiculobullous lesions [52].

The scalp is the main site of follicular CLP or LPP as described above.

LP causes nail lesions in 1–10% of adult cases (Figures 1(h) and 1(i)). Nail LP is more commonly seen in children [53]. One study reported a prevalence of 19% in a cohort of 100 childhood LP cases from India [22]. Tosti and coworkers noted that nail LP may be underdiagnosed in children because of the fact that isolated nail lesions are more common in this age group [53]. Likewise, Kanwar and De suggested that the general hesitation of dermatologists to perform nail biopsies on children may be another reason for underdiagnosis of the disease in this age group [22]. Fingernails are more commonly involved than toenails [54]. Although it can affect both the nail matrix and the nail bed, the diagnosis of classic nail LP should be based on destruction of the nail bed with longitudinal fissuring and ridging of the nail plate, brittleness (onychorrhexis) and spontaneous separation of the nail plate (onycholysis) [53, 55, 56]. Nail involvement may irreversibly deform or destroy the nails. Dorsal pterygium is one of the characteristic findings and may be present in the classic form [53, 57]. Twenty-nail dystrophy is characterized by nail coarseness affecting all fingernails and toenails because of excessive longitudinal ridging (trachyonychia). This clinical presentation is observed in LP as well as other diseases that involve the nail matrix such as alopecia areata, psoriasis, eczema, and pemphigus vulgaris [53]. In rare circumstances, localized distal subungual hyperkeratosis with multinucleated cells can arise (onychopapilloma) [58]. Additionally, melanonychia can be induced with resolution of the lesions [59]. The lunula may be irregularly red in a focal or disseminated pattern due to the associated inflammation. In the toenails, remarkable thickening may be noted and can be mistaken with yellow nail syndrome. Furthermore, erosive nail LP may rarely occur with painful erosions and consequent scarring [54]. Another rare variant, idiopathic atrophy of the nails, can cause rapid diffuse nail atrophy and pterygium of the nails and can ultimately lead to permanent anonychia [54].

#### 3.3.2. Mucosal LP

Mucosal LP more commonly involves the oral mucosa but can also affect the vulvovaginal area. LP can rarely manifest in the esophagus, larynx, and conjunctiva [60].

**Figure 2:**
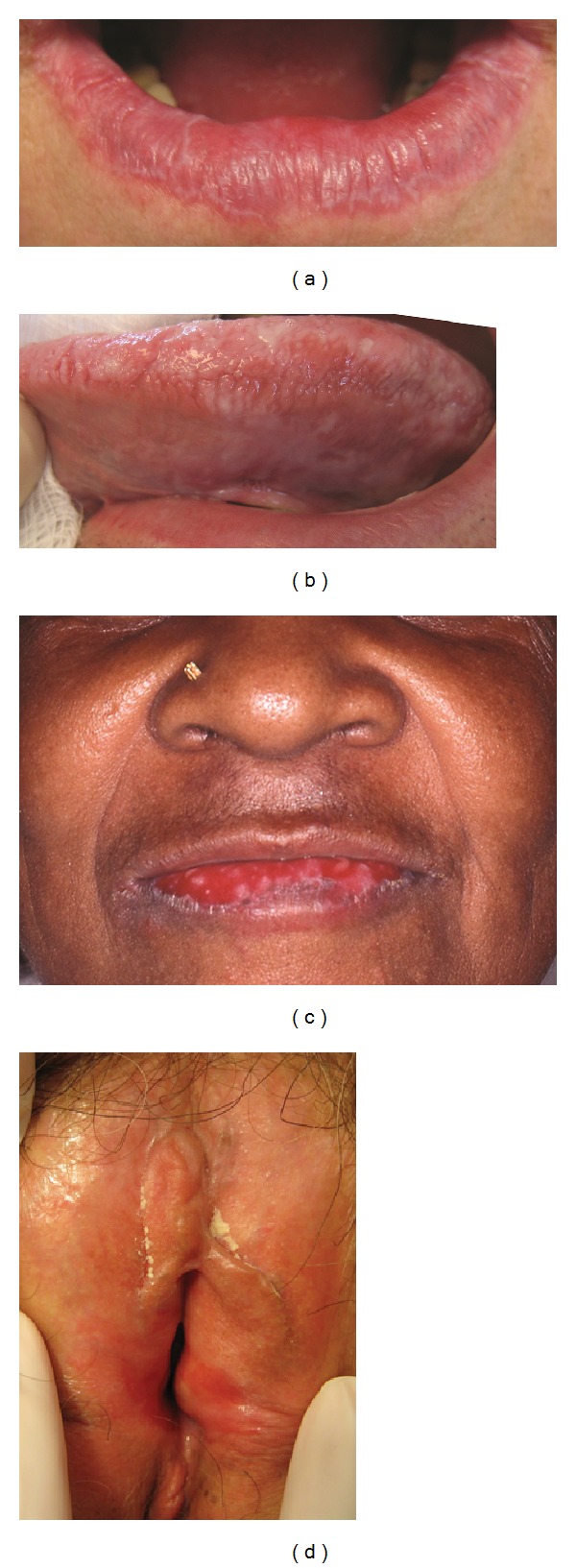
(a) Reticular OLP: reticulated white striae of the lower lip proper; (b) plaque-type OLP: confluent white reticulated plaques of the lateral tongue; (c) erosive OLP: superficial erosions and white striae of the lower lip proper (courtesy of Dr. Peter Lynch); (d) vulvar LP: superficial erosions at the vulvar introitus with fusion of the labia minora and vaginal stenosis; Figures 1(a) and 1(b) are reprinted with permission from [50]. OLP, oral lichen planus.

*OLP*. OLP has several clinical subtypes including reticular, erosive, atrophic, papular, plaque-like, and bullous subtypes. Figures 2(a)–2(c) illustrate different subtypes of OLP. The buccal mucosa is typically involved in 80–90% of OLP cases. The Koebner phenomenon is not only present in CLP but can also occur in the setting of OLP. Eisen [20] suggested that the mechanical trauma of dental procedures, cigarette smoking, mucosal trauma from sharp cusps, and oral habits such as lip chewing are Koebnerogenic factors that can exacerbate OLP. Reticular OLP, the most common subtype, is usually asymptomatic. Hence, it is often diagnosed during a routine oral examination. Reticular OLP is characterized by white lacy streaks surrounded by well-defined erythematous borders. This pattern is less evident on the dorsum of the tongue [61]. In some patients, reticular OLP may eventually progress to the more severe subtypes such as the erosive form. Papular OLP is characterized by small white pinpoint Papules that can be easily missed as they are small and asymptomatic. It is referred to as the initial and transient phase of OLP [62]. That is why it is a rare diagnosis [63, 64]. In plaque-like OLP, large, homogenous white patches are characteristic. Plaque-like OLP and leukoplakia have similar clinical presentations and therefore leukoplakia must always be ruled out [64]. This variant is more prevalent in tobacco smokers [62]. The existence of plaque-like lesions is an indicator of a poor prognosis and a lesser likelihood of remission [62]. Erosive OLP, the most advanced subtype, can clinically present as atrophic or erythematous ulcerations and erosions of the mucosa and faint radiating white striae. The associated ulcers are sometimes covered with a pseudomembrane. Typically, it has a multifocal pattern of distribution. It is clinically important because the lesions can be quite painful and therefore it may negatively affect the patient's quality of life. The symptoms may range from discomfort to severe, painful episodes. Involvement of the dorsum of the tongue might cause dysgeusia [16]. The atrophic subtype is common presentation that has similarities to the erosive subtype with more prominent atrophic lesions on a background of erythema and radiating white striae at the margins. Thus, some experts combine the two entities and name it atrophic-erosive lichen planus. This subtype is more common in older OLP patients [62]. Atrophic OLP primarily affects the attached gingiva [64]. The buccal mucosa can also be involved, particularly in the posteroinferior areas adjacent to the second and third molar teeth [65]. In rare instances, OLP may present with bullous lesions. Lourenço and colleagues suggested a rare form of OLP as “Lichen planus sialadenitis,” a mucosal analog of LPP due to lymphocyte infiltration in the salivary gland ducts [66].

*Vulvovaginal LP*. Vulvar LP can affect peri- or postmenopausal women with rare occurrence in children [67]. Vulvovaginal LP has a similar pattern to OLP.  Figure 2(d) illustrates vulvar LP. It has three major subtypes: erosive, papulosquamous, and hypertrophic [68]. Erosive vulvar LP is the most frequent subtype, involving the mucous membranes exclusively [69]. The most significant sequelae of chronic erosive vulvar LP are scarring, which can result in resorption of the labia minora (agglutination) and clitoral hood, with subsequent clitoral burying (synechiae, 68%), stenosis of the introitus (59%), and potentially total obliteration of the vagina. The associated scarring and adhesions affecting the vagina may interfere with sexual intercourse [70, 71]. It is essential to consider the patient's psychological status and quality of life, which can be deeply affected by the disease. Sexual abstinence or dyspareunia was present in 46 out of 58 patients diagnosed with genital erosive LP referred to a specialized center in Norway over a 7-year period [72]. It is also beneficial to document the extent and location of the lesions in the vulva and examine other mucocutaneous sites of LP at each visit [73]. In addition to dyspareunia, vulvar LP can lead to symptoms of intense pruritus with chronic vaginal discharge, burning, and postcoital bleeding [72, 74]. The presentation of papulosquamous and hypertrophic vulvar LP is similar to their corresponding OLP subtypes. Furthermore, 43%–100% [60, 70, 73] of vulvar cases may have concomitant oral involvement, whereas about 25% of OLP patients may have vulvar involvement [20]. The coexistence of oral and genital lesions is known as vulvovaginal-gingival syndrome [75]. Skin lesions are as frequent as 17%–22% in this syndrome [60, 73].

*Esophageal LP*. LP of the esophagus is a rare presentation of the disease. It is an underdiagnosed and underreported entity with a sexual predilection in women [76]. In the majority of cases, esophageal LP may be accompanied by other mucosal lesions or less commonly concomitant with cutaneous manifestations. It may also present as an isolated disease in its initial presentation (20%). Ultimately, extraesophageal lesions can be found in almost all patients with esophageal LP [76]. The proximal esophagus is affected in 90% of cases with or without distal involvement. In may cases, there is a significant delay between the onset of symptoms and detection of esophageal involvement. Thyroid dysfunction is the most common associated disorder [76]. In a case-control study, superficial gastritis was significantly more common in LP patients than healthy controls [77]. Therefore, it is important to consider upper GI endoscopy particularly in LP patients with complaints of dysphagia, odynophagia, weight loss, or other esophageal symptoms and those with involvement of other mucosal surfaces [78].

*Ocular LP*. LP may be accompanied by significant ophthalmologic problems including a decrease in tear production [79]. More than one third of LP patients have blepharitis [79]. Şanli and coworkers also noted a lower number of goblet cells in the conjunctival epithelium of LP patients, when compared to controls [79]. Ocular involvement may also cause mild to moderate xerophthalmia and occasionally cicatricial conjunctivitis [79, 80]. Conjunctival involvement can start with white streaks involving the palpebral conjunctiva [80]. In a case series of 9 LP patients with ophthalmological signs, 7 cases had vulvovaginal-gingival syndrome and all patients developed subepithelial fibrosis and lacrimal duct stenosis [81]. Webber et al. recommended ophthalmologic evaluation and examination of the lacrimal duct puncta in erosive LP patients [81]. Other ophthalmologic signs include eyelid lesions [82, 83], keratouveitis [80], keratoconjunctivitis sicca [80], punctate epithelial erosions [80, 84], corneal ulceration/scarring [84], and dysplastic conjunctival lesions resembling ocular surface squamous neoplasia [85]. LP was also reported as chronic keratoconjunctivitis with diffuse conjunctival hyperemia, subconjunctival fibrosis, and forniceal symblephara with foreshortening of the lower fornix in the isolated ocular form [86]. This form of the disease can be a diagnostic challenge [87]. Biopsy for histopathologic examination and immunofluorescence studies is the only way to differentiate ocular LP from other causes of irreversible scarring keratoconjunctivitis [86]. The presence of subepithelial fibrinogen is not necessarily inclusive of any particular eye disease on immunofluorescence analysis, but a fragmented and shaggy subepithelial fibrinogen layer in the conjunctiva is indicative of LP [88, 89]. It is essential to diagnose and treat such disease quickly and efficiently to avoid the dire consequences of blindness [90].

*Laryngeal LP*. Involvement of the larynx is extremely rare. In fact, few patients with laryngeal LP have been reported [91–93]. The first case was a 57-year-old Caucasian man with isolated laryngeal LP and complaints of hoarseness [91]. The second case was an 18-year-old Pakistani man with mucosal LP in the mouth, conjunctivae, and larynx [92]. Kunelskaya Ya. and Arievich reported the largest case series of laryngeal involvement in 8 mucosal LP patients. They noted involvement of epiglottis and aryepiglottic folds but no involvement of vocal cords. The lesions were larger in the epiglottis region than other parts of the larynx [93].

Table 1 addresses the most common sites of involvement for LP subtypes.

### 3.4. Diagnosis

The diagnosis of LP is based on the clinical presentation and should be confirmed by biopsy, if suspected. Histopathology is often conclusive, but in vesiculobullous CLP or erosive OLP, direct immunofluorescence (DIF) studies can be an integral step in differentiating between LP and other diseases [16]. DIF typically demonstrates globular IgM deposition at the dermal-epidermal junction in LP [94]. Although the existence of fibrin deposition at the mucosal submucosal interface and within vessels and the presence of colloid bodies is highly sensitive for a diagnosis of LP, it lacks specificity [95]. Indirect immunofluorescence and enzyme-linked immunosorbent assays can also be helpful in reaching a diagnosis [16].

#### 3.4.1. Histopathology

LP is characterized by lichenoid interface dermatitis. The classic histopathological features include a dense, continuous, and band-like lymphohistiocytic infiltrate at the dermal-epidermal junction and in the upper dermis. Characteristically, the infiltrate disguises the dermal-epidermal junction and makes it difficult to recognize the basal layer at the early stages of the disease [96]. Epidermal changes in LP lesions include irregular epidermal hyperplasia with a jagged “sawtooth” appearance, compact hyperkeratosis or orthokeratosis, foci of wedge-shaped hypergranulosis, basilar vacuolar degeneration, slight spongiosis in the spinous layer, and squamatization. The dermal papillae between the elongated rete ridges are frequently dome shaped [97]. Necrotic keratinocytes can be observed in the basal layer of the epidermis and at the dermal-epidermal junction. Eosinophilic remnants of anucleate apoptotic basal cells may also be found in the dermis and are referred to as “colloid or civatte bodies” [1]. Whickham striae are usually seen in the areas of hypergranulosis [1]. Vacuolar degeneration at the basal layer may be noted leading to focal subepidermal clefts (Max Joseph spaces) [98]. Squamatization occurs as a result of maturation and flattening of cells in the basal layer [1]. It happens in areas of marked hypergranulosis with prominence of the sawtooth pattern of rete ridges [94]. Wedge-shaped hypergranulosis can occur in the eccrine ducts (acrosyringia) or hair follicles (acrotrichia) [99]. In the hypertrophic subtype, the associated hyperkeratosis, parakeratosis, hypergranulosis, papillomatosis, acanthosis, and hyperplasia markedly increased with thicker collagen bundles forming in the dermis [94, 99]. Moreover, the rete ridges are more elongated and rounded as opposed to the typical sawtooth pattern [94]. In atrophic LP, loss of the rete ridges and dermal fibrosis is prominent [99]. In vesiculobullous LP, the disease progression is quicker. Hence, some of the distinctive features such as hyperkeratosis, hypergranulosis, or dense lymphocytic dermal-epidermal infiltrate may not be present [99]. LP lesion may resolve with residual hyperpigmentation caused by a persistent increase in the number of melanophages in the papillary dermis [96, 100].

In classic LPP, the bandlike lymphocytic infiltrate is initially contained in the peribulge area including the infundibulum and isthmus with sparing of the lower segment of the hair follicle. The same follicular segments may exhibit orthokeratosis, hypergranulosis, and follicular plugging [96]. The interfollicular epidermis is rarely affected [43]. LPP leads to permanent hair loss due to the involvement of hair follicle stem cells in the bulge. Fibrotic tissue can progressively replace the hair follicles [101].

In mucosal lesions, the epithelial changes are less specific. The rete ridges do not exhibit the characteristic pronounced sawtooth pattern because normal oral mucosa exhibits parakeratosis with no granular layer [102]. Hence, OLP lesions rarely exhibit orthokeratosis. OLP lesions are likely to be more atrophic than acanthotic as compared to CLP [96]. Contrary to CLP and OLP, histolopathological findings in genital erosive LP are less specific and often inconclusive [70, 73].

#### 3.4.2. Differential Diagnosis

The differential diagnosis of LP is fairly broad and summarized in Table 2. An important entity in the differential diagnosis of LP is lichenoid drug reactions, which can virtually be indistinguishable from cutaneous LP both clinically and histopathologically. Typically, lesions have a photodistribution in the absence of oral mucosal involvement. The most commonly implicated drugs are summarized in Table 3. Lichenoid drug reactions characteristically exhibit parakeratosis, a dermal eosinophilic infiltrate, and a perivascular lymphocytic infiltrate affecting the reticular dermis. Epidermal changes are less common in lichenoid drug eruptions when compared to classic LP [94]. However, a higher concentration of necrotic keratinocyte and eosinophils in the infiltrate can be helpful in distinguishing lichenoid drug reaction from cutaneous LP [103]. A lengthy interval between the commencement of drug therapy and the onset of lesions does not exclude a diagnosis of lichenoid drug reaction [16]. Resolution of the lesions often occurs within weeks to months after discontinuation of the offending drug [104].

Lichenoid contact reaction is an important consideration in the differential diagnosis of LP. Patch testing and challenge testing can be utilized to properly identify the causal allergen. As suggested in other patch test studies, one should interpret positive patch test results to any particular allergen in the context of its clinical relevance. In a retrospective cohort study of patients with contact stomatitis or contact mucositis, 46/198 patients were diagnosed with OLP and had undergone patch testing. Fourteen out of forty-six patients (40%) had identifiable contact hypersensitivity [105]. The main triggering contact allergens involved in lichenoid contact reactions are shown in Table 4. Metals can induce or aggravate lichenoid contact reactions including silver-mercury amalgam fillings and other metals containing dental restorative materials [106]. In a prospective study from Basque, such lichenoid reactions were limited to old and corroded dental fillings [107]. Even patients with negative patch test results are likely to benefit from removing corroded restorations simply by omitting the mucosal irritation induced by them as a result of Koebner's phenomenon [108]. Food flavorings can also induce lichenoid reactions including cinnamon, cinnamonic aldehyde, and spearmint oil present in foods and dentifrices [109, 110]. A rare lichenoid form of mycosis fungoides (MF) can mimic CLP lesions. Lichenoid MF has more eosinophils, mast cells, lymphocytic nuclear atypia, and more basilar epidermotropism as compared to CLP [111]. Lichenoid MF is extremely pruritic and has a poor prognosis. Therefore, prompt diagnosis and management is crucial [111].

Resolving ashy dermatosis can be considered in the differential diagnosis of hyperpigmented CLP lesions. Both have similar histopathologic features. However, they can be differentiated from each other by their color (blue-gray/ashy-brown for ashy versus black-brown/violet-blue for LP), the absence of erythematous borders in LP pigmentosus, and the absence of itching in ashy dermatosis [112].

Lichen nitidus may be difficult to clinically distinguish from generalized LP with a dense lymphohistiocytic infiltrate on histopathology [113].

Differentiating between pseudopelade of Brocq and scarring classic LPP can be challenging as they both have multifocal involvement of the vertex scalp although LPP presents more frequently with perifollicular erythema and follicular keratotic plugs than pseudopelade of Brocq [43, 114]. There is an ongoing debate as to whether pseudopelade of Brocq is a subtype of LPP [47].

Lichen striatus has a clinical presentation similar to linear LP. It presents as unilateral linear erythematous itchy Papules in young adults and children. It is histopathologically differentiated from linear LP by exhibiting more prominent parakeratosis and spongiosis [115].

Distinguishing between the cicatricial alopecia induced by LPP and discoid lupus erythematosus (DLE) can be challenging. Unlike DLE, LPP often spares the interfollicular epidermis [42]. Dermatoscopic examination can also be helpful. Perifollicular scale and branching capillaries are distinguishing dermatoscopic signs for LPP and DLE, respectively [116].

Granuloma annulare does not present with scale or Wickham striae, but it may resemble annular LP [34].

Vesiculobullous CLP and LP pemphigoides are difficult to differentiate. LP pemphigoides can be clinically distinguished by a more generalized distribution, more extensive blistering, and a more chronic course [117].

Blistering diseases and lupus erythematous are on the list of differential diagnoses of OLP. The differential diagnosis of OLP from other diseases is particularly difficult in the non-reticulated forms often necessitating biopsy and DIF studies [16]. Erosive/ulcerative OLP should be distinguished from mucous membrane pemphigoid. The presence of whitish hyperkeratotic striations and skin involvement are important differentiating features [118]. The DIF pattern of a pemphigoid lesion will most often include linear IgG and C3 and rarely, IgM at the basement membrane level.  Table 5 presents the DIF patterns of the differential diagnoses of LP.

Vulvar mucosal LP can be misdiagnosed as lichen sclerosis or vulvovaginal blistering diseases. The classic Wickham striae and histopathologic examination can be helpful in making the distinction.

### 3.5. Immunopathogenesis

LP is a T-cell-mediated autoimmune disease. Inflammatory cells involved in this process consist of T helper and T cytotoxic lymphocytes, natural killer (NK) cells, and dendritic cells. T-cell activation is central to the pathogenesis of LP. Cytotoxic T-cell infiltration into the epithelium results in apoptotic basal keratinocytes. Theoretically, it may be induced by CXCR3 and CCR5 mediated signaling pathways initiated by both T-cells and keratinocytes [119]. In the early stages, T-cells predominantly home in the deeper layers of the epidermis and at the dermal-epidermal junction [119]. CCR5 related chemokines as well as CXCR3-targeting chemokines are significantly overexpressed in LP lesions in concert with the increased trafficking of mononuclear cells to the interface region [120, 121]. This correlation or coincidence suggests that both keratinocyte induced- and self-recruiting mechanisms are involved in T-cell migration within LP lesions. Furthermore, Langerhans cell recruitment is induced by CCR6 related chemokines [119]. Activated T-cells stimulate the T helper type 1 (Th1) response, thus resulting in keratinocyte removal by immune cells [120].

The TH1 dominance in LP patients is partly regulated through toll-like receptor (TLR) 2 upregulation [120]. TLR is mainly involved in innate immunity but can also trigger adaptive immunity. TLR2 activation is known to induce TH1 activation. However, there is a contrasting result from a study suggesting downregulation of TLR2 and upregulation of TLR4 in OLP patients [122]. Moreover, in OLP patients, TLR4 and TLR9 are upregulated in the superficial and basal layers, respectively [123].

NK cells can migrate to LP lesions more frequently than to healthy skin [124]. They may contribute to the pathogenesis of LP due to their cytotoxic activity and their ability to secrete proinflammatory cytokines [124].

One of the main pathogenetic mechanisms of LP is increased apoptosis of keratinocytes and decreased apoptosis of T-cells [125]. Activated cytotoxic T-cells can upregulate the Fas ligand and induce keratinocyte apoptosis in the suprabasal cell layer by binding to Fas on the surface of keratinocytes [126]. NK cells and cytotoxic T-cells may also induce apoptosis via the granzyme B/perforin pathway. This autodestructive immunologic mechanism is more abundant in OLP than CLP [127]. The altered immune response results in apoptosis of basilar keratinocytes and ultimately leads to liquefaction of the entire basal layer. These apoptotic changes may also be a reflection of the disease activity [128].

Ragaz and Ackerman noted an increased number of Langerhans cells in the epidermis very early in the disease process [99]. These cells function to present the autoantigens or foreign antigens to T-cells prior to T-cell activation. Langerhans cells are more prevalent in OLP lesions than healthy oral mucosa and the degree of migration of these cells from the superficial layers to the basal layer may be a predictor of disease chronicity [129].

## 4. Etiology and Risk Factors

### 4.1. Immunogenetic Factors

LP is a complex disease and thus can be caused or triggered by genetic malfunction and/or environmental factors. The existence of familial cases of LP may suggest a possible genetic predisposition [130, 131]. Gene polymorphisms of different HLA markers as well as the inflammatory cytokines and chemokines have been associated with the presence of LP (Table 6). The causality of these polymorphisms, although unclear, supports the autoantigen hypothesis.

### 4.2. Clinical Factors

Associated factors and disease conditions seen in LP include but are not limited to stress/anxiety, hepatitis C virus (HCV), autoimmune diseases, internal malignancies, dyslipidemia, and viral infections. Anxiety is a well-established risk factor or accompanying factor in LP patients [132]. Some studies have indicated that stressful events can induce LP lesions in otherwise healthy individuals. In a case-control study, more than 67% of LP patients experienced a stressful event while about 21% of matched healthy controls experienced such events [133]. Other studies more or less indicate a similar trend for stress, anxiety, and depression [134, 135].  Table 7 summarizes the coexistence of some clinical conditions and LP.

We performed a meta-analysis to find the potential association between LP and HCV including all studies in previously published meta-analyses [136–138] and new studies [139–143], which investigated the prevalence of HCV in LP patients compared with a control population. Based on data pooling of 64 studies, LP patients have 5.58 times the odds of having concurrent HCV infection than the control population (95% CI: 3.72–8.38, *P* < 0.05).

In an attempt to investigate the role of HPV in the pathogenesis of OLP, Syrjänen et al. [144] performed a meta-analysis. They found statistically remarkable and significant odds ratios for HPV, in general, and HPV-16 (5.12 (95% CI: 2.40–10.93) and 5.61 (95% CI: 2.42–12.99), resp.). This finding suggests that HPV has a higher prevalence in OLP patients than the normal population; it may have a role in the malignant transformation of OLP lesions [145]. Erosive OLP and hypertrophic CLP are considered as the main subtypes with malignant potential [20, 146].

A systematic search revealed three studies [189–191] that compared the prevalence of the transfusion transmitted virus or Torque tenovirus (TTV) in LP versus disease-control patients or healthy volunteers. TTV is the only member of Anelloviridae [192]. After performing a meta-analysis using two studies [190, 191], there was no significant difference between LP and healthy control groups (odds ratio of 1.13 [95% CI: 0.67–1.89], *P* > 0.05). Fehér et al. [190] recently showed that genogroup 1 TTV—not TTV in general—is significantly more common in OLP patients (10.1%) when compared to the control group (1.4%). This group hypothesized that genogroup 1 TTV is correlated with the immuno-inflammatory response in OLP patients. They also evaluated the subtypes and genotypes of TTV in the aforementioned patients and noted some differences in OLP lesions and normal skin samples from the same patients [193].

While the varicella zoster virus is rare in OLP patients [194, 195], a recent study noted a significant role for the virus in zosteriform LP but not in linear LP [196].

LP patients are at increased risk of developing dyslipidemia, with an adjusted odds ratio of 2.85 (95% CI, 1.33–5.09; *P* = 0.001) [197]. However, patients with hyperglycemia and/or hypertension are not at risk [197].

## 5. Quality of Life

Quality of life questionnaires are easy and practical tools to quantify the impact of a disease based on the patient's perception. There are a large number of general and dermatology-related quality of life questionnaires including the Dermatology Life Quality Index (DLQI), which is a widely used dermatology specific quality of life instrument [276]. LP was comparable to psoriasis with regard to Dermatology Life Quality Index (DLQI) scores of 9.60 ± 7.32 versus 9.50 ± 6.10, respectively. Additionally, OLP patients had a significantly higher DLQI score when compared to CLP patients (13.27 ± 8.05 versus 7.47 ± 6.11) [277]. The Oral Health Impact Profile (OHIP) is a 49 item quality of life questionnaire to evaluate the social impact of oral disorders which is based on theoretical hierarchy of oral health outcomes [278]. OLP was found to have a significant impact on psychological discomfort and social disability, using OHIP 49 item questionnaire [279]. Furthermore, OLP seems to have a higher impact on patients' quality of life than recurrent aphthous stomatitis but lower impact as compared to oral bullous diseases [280]. There is also a correlation between increase in pain evaluated by visual analogue scoring and poor oral health-related quality of life in patients with LP [281]. Patients with erosive OLP or generalized LP may have poor quality of life because of the associated pain and discomfort.

### 5.1. Prognosis

Typically, CLP lesions resolve within 6 months to a year. However, the hypertrophic variant, if left untreated, tends to persist for years. Untreated reticular OLP has a chronic or progressive nature, usually without complete resolution. LP can also have a recurrent pattern. Patients with erosive LP may experience changes in location and severity of the disease with waxing and waning cycles of concurrent healing and lesion formation. Although generalized LP tends to heal faster than other variants, it has a greater likelihood of relapse [2]. LPP can be progressive in its course with destruction of hair follicles leading to atrophic cicatricial alopecia [43].

### 5.2. Carcinogenic Transformation

The carcinogenic potential of OLP lesions has been a longstanding topic of debate. When loss of heterozygosity and microsatellite instability were investigated as two of the main indicators for malignant transformation, OLP was not different from benign fibroma, but it was significantly different from low-grade oral dysplasia, high-grade oral dysplasia, and oral SCC [282]. Vered et al. clearly suggested a widespread proinflammatory response rather than a protumorigenic response [283]. Similar to other malignancies, the elderly are at increased risk for developing SCC [284] and tend to have more severe forms of the disease [285].

The epidemiology surrounding OLP and its inherent risk of oral squamous cell carcinoma remains poorly defined. A question, that has yet to be answered, is whether the associated risk of malignant transformation is intrinsic to the OLP lesions or as a result of the patient's immune response or genetic background. There is no proper surrogate biomarker for OLP malignant transformation. Shi et al. suggested the co-expression of podoplanin and ATP-binding cassette transporter G2 (ABCG2) as a higher prognostic marker with a significant odds ratio (25.24, 95% CI: 4.48–142.27, *P* < 0.001) [284]. Unfortunately, podoplanin is not sensitive or specific, as it can be expressed even in normal skin [286]. Additionally, ABCG2 is expressed in various organs and has a major role in tissue repair [287] in addition to chemoresistance of different tumors [288]. Another marker, c-Jun, a member of activating protein-1 transcription factor family, was present in 11 out of 12 LP patients and in all SCC patients while not expressed remarkably in normal patients. These transcription factors contribute to cell proliferation and thus they may be indications of LP's carcinogenic potential [289]. These reports should be interpreted cautiously due to the small sample size and lack of prospective follow-up. The literature is still inconclusive in this regard except for the fact that it seems improbable that OLP is inherently carcinogenic [290].

The malignant transformation rate of vulvar LP and esophageal LP seems to be 1.1% [73] and 5.5% [76], respectively. In a cohort study with no healthy controls and without correcting the odds ratio for premalignant viral diseases, 8 of 327 OLP patients (2.4%) developed oral SCC in previously affected areas. Interestingly, this was not correlated with immunosuppressive therapy in those patients [291]. The transformation ratio for OLP lesions varies from 0.8% in an American population [20] to more than 5% in an Italian cohort [292]. This ratio appears to be no more than 1% for OLP over 5 years [293]. Nonetheless, this estimated rate does not match the prevalence of OLP and oral cancer. Indeed, an OLP prevalence of 1% and a transformation rate of 0.2% per year would mean that almost every single oral SCC should arise from an OLP lesion. Hence, this theory was refuted as few accompanying OLP lesions were detected in oral SCC patients [293–296]. Further investigation of whether chronic LP is a premalignant condition would necessitate large-scale prospective cohort studies with a long-term follow-up period.

## 6. Conclusions

LP is a T-cell mediated disease. The prevalence of LP is less than 5% with no evident sexual predilection. The chronic and often erosive nature of LP can have detrimental effects on patients' quality of life. Erosive-ulcerative OLP lesions tend to become painful and chronic and vulvar LP may interfere with sexual intercourse and can be associated with significant psychological and physical morbidity. HCV and HPV are more prevalent in LP patients compared to the normal population. The carcinogenic potential of OLP lesions remains debatable.

## Figures and Tables

**Table 1:** The most common sites of involvement in LP based on subtypes.

Subtypes	Most common sites of involvement
CLP	
Actinic	Sun-exposed areas such as face, V-chest, hands
Annular	Male genitalia (penis, scrotum), axilla, groin folds
Atrophic	All parts of body especially lower extremetities
Erosive	Soles of feet
Follicular	Scalp
Guttate	Trunk
Hypertrophic	Anterior leg, ankles, and interphalangeal joints
Linear	Leg-excoriated area
Papular	Flexor surfaces (the main initial presentation)
Bullous	Feet
Pigmentosus	Sun exposed areas such as face, V-chest, hands
Pigmentosus-inversus	Intertriginous and flexural areas
Nail involvement	Fingernails and toenails
Palmoplantar involvement	(1) Malleoli (2) Soles (internal plantar arch)
Lichen planopilaris	(1) Vertex of scalp (classic type) (2) Frontal of scalp (FFA type)

Mucosal LP	
Oral	
Reticular	(1) Buccal mucosa and mucobuccal folds (2) Lateral and dorsal tongue (3) Gingiva and lips
Atrophic	Attached gingiva
Hypertrophic	Buccal mucosa
Erosive	(1) Lateral and ventral portions of tongue (2) Buccal mucosa
Bullous	Posterior and inferior areas of buccal mucosa
Plaque-like	Dorsum of the tongue and buccal mucosa
Vulvovaginal	
All subtypes	Vaginal introitus, clitoris, clitoral hood, labia minora, and majora, vagina
Esophageal	(1) Proximal esophagus (2) Proximal and distal esophagus (3) Distal esophagus, strong possibility of concomitant mucosal involvement (80%)

FFA: frontal fibrosing alopecia, CLP: cutaneous lichen planus, FFA: frontal fibrosing alopecia, LP: lichen planus.

**Table 2:** LP differential diagnosis.

CLP	
Papular classic	Psoriasis, chronic cutaneous lupus erythematosus, lichen simplex chronicus, graft-versus-host disease, secondary syphilis, pityriasis rosea, lichenoid mycosis fungoides
Annular	Granuloma annulare, tinea
Linear	Lichen striatus, inflammatory linear verrucous epidermal nevus (ILVEN), linear psoriasis, linear Darier-White disease, nevus unius lateris
Hypertrophic	Psoriasis, prurigo nodularis, lichenoid cutaneous amyloidosis, Kaposi sarcoma, lichen simplex chronicus, stasis dermatitis
Vesiculobullous	Lichen planus pemphigoides, bullous pemphigoid, pemphigus vulgaris
LP pigmentosus	Ashy dermatosis
Atrophic	Lichen sclerosus et atrophicus, lupus, ashy dermatosis
Generalized	Lichen nitidus, drug eruptions, guttate psoriasis, viral exanthems

LP of skin appendages	

Follicular CLP (LPP)	Lichen spinulosus
LPP induced cicatricial alopecia	Pseudopelade of Brocq, follicular degeneration syndrome, androgenetic alopecia, chronic cutaneous lupus erythematosus
Nail CLP	Brittle nails, lichen striatus, graft versus host disease, systemic amyloidosis, trauma, dyskeratosis congenital
Idiopathic atrophy of nails	Hereditary anonychia, impaired peripheral circulation, epidermolysis bullosa
Trachyonychia	Alopecia areata and psoriasis
Toenail LP	Yellow nail syndrome
Erosive nail LP	Blistering disease nail involvement

Mucosal LP	

OLP	Leukoplakia, candidiasis, erythema multiforme, pemphigus vulgaris, bullous pemphigoid, lichen sclerosus, secondary syphilis, bite trauma, lichen sclerosus et atrophicus, lupus, ashy dermatosis
Vulvar LP	Lichen sclerosis, valvovaginal blistering diseases

CLP: cutaneous lichen planus, LP: lichen planus, LPP: lichen planopilaris, OLP: oral lichen planus.

**Table 3:** Drugs responsible for lichenoid reaction.

**Anticholinergics** (i) Solifenacin **Anticonvulsants** (i) Carbamazepine (ii) Oxcarbazepine (iii) Phenytoin (iv) Valproate sodium **Antidiabetics** (i) Chlorpropamide (ii) Glyburide (iii) Glipizide (iv) Insulin (v) Tolazamide (vi) Tolbutamide **Antidiarrheals** (i) Bismuth **Antigout** (i) Allopurinol **Antihistamine drugs** (i) Cinnarizine (ii) Iodides **Anti-inflammatory drugs** (i) Aspirin (ii) Diflunisal (iii) Ibuprofen (iv) Indomethacin (v) Leflunomide (vi) Mesalamine (vii) Naproxen (viii) Rofecoxib (ix) Sulindac (x) Sulfasalazine (xi) Tolbutamide **Antimalarials** (i) Chloroquine (ii) Hydroxychloroquine (iii) Pyrimethamine (iv) Quinidine (v) Quinine **Antimicrobials** *Antibiotics* (i) Para-aminosalicylic acid (ii) Sulphamethoxazole (iii) Tetracycline *Antimycobacterials* (i) Aminosalicylate sodium (ii) Dapsone (iii) Ethambutol (iv) Isoniazid (v) Rifampin (vi) Streptomycin *Antifungals* (i) Amphotericin B (ii) Griseofulvin (iii) Ketoconazole *Antihelminthic* (i) Levamisole	**Cardiovascular drugs** (i) Atenolol (ii) Captopril (iii) Doxazosin (iv) Diazoxide (v) Enalapril (vi) Labetalol (vii) Metoprolol (viii) Methyldopa (ix) Nebivolol (x) Nicorandil (xi) Nifedipine (xii) Prazosin (xiii) Procainamide (xiv) Propranolol (xv) Quinidine (xvi) Terazosin **Diuretics** (i) Chlorothiazide (ii) Furosemide (iii) Hydrochlorothiazide (iv) Spironolactone **Immunomodulatory and biological therapies** (i) Adalimumab (ii) Dactinomycin (iii) Etanercept (iv) Gold salts (v) Imatinib mesylate (vi) Infliximab (vii) Interferon-*α* (viii) Penicillamine (ix) Tacrolimus **Lipid lowering drugs** (i) Gemfibrozil (ii) Orlistat (iii) Pravastatin (iv) Simvastatin **Metals** (i) Arsenic **Psychiatricdrugs** (i) Antipsychotics (Chlorpromazine, Levomepromazine, Methopromazine, Thioridazine) (ii) Benzodiazepines (Lorazepam) (iii) Imiquimod (iv) Lithium (v) Selective serotonin reuptake inhibitor (Escitalopram) (vi) Tricyclic antidepressants (Amitriptyline, Imipramine) **Retinoids** (i) Isotretinoin
**Antiparkinsoniandrugs** (i) Trihexyphenidyl **Antiretrovirals** (i) Zidovudine **Chemotherapy drugs** (i) Hydroxyurea	**Other medications** (i) Amiphenazole (ii) Clopidogrel (iii) Penacillamine (iv) Palifermin (v) Mercapto-propionylglycine (vi) Misoprostol (vii) Nandrolene (viii) furyl-propionate (Demelon) (ix) Norflex (x) Omeprazole (xi) Pyrithioxin (xii) Sildenafil (xiii) Tiopronin

Modified from Schlosser [16].

**Table 4:** Possible contact allergens to induce lichenoid contact reactions.

Dental restoration/ crowning materials	Beryllium, cobalt, copper, chromium, gold, indium, mercury, nickel, palladium, silver, tin, zinc, composite resins, ceramics (porcelain), ethylene glycol dimethacrylate, titanium

Flavorings	Menthol oil, peppermint, cinnamon, spearmint, balsam of Peru, vanillin, jasmine absolute, lemon oil, eugenol

Modified from Schlosser [16].

**Table 5:** The pattern of DIF results of potential differential diagnoses of lichen planus.

Differential	DIF pattern
Lichen planus	Globular IgM and fibrin deposition at BMZ
Aphthous ulcers	Negative
Bullous pemphigoid	Linear C3, IgG at BMZ, less common IgA
Chronic ulcerative stomatitis	Speckled or granular perinuclear IgG in the lower third and basal layer of epithelium
Dermatitis herpetiformis	Granular IgA at BMZ with concentration at the papillary tips
Discoid lupus erythematosus	Linear band or continuous granular IgG, IgA, IgM, and C3 at BMZ
Epidermolysis bullosa acquisita	Linear IgG and C3 at BMZ
Erythema multiforme	Negative
Hailey-Hailey disease	Negative
Lichen nitidus	Negative
Linear IgA bullous dermatosis	Linear IgA at BMZ, less common IgG, IgM, and C3
Mucous membrane pemphigoid	Linear IgG and C3 at BMZ, less common IgA, IgM, and/or fibrin
Paraneoplastic pemphigus	Intercellular IgG and C3 with or without BMZ involvement
Pemphigus vulgaris	Intercellular IgG (IgG1 and IgG4) in “chicken wire pattern,” less common C3 and IgM
Systemic lupus erythematosus	Linear band or continuous granular IgG, IgA, IgM, and C3 at BMZ

BMZ: basement membrane zone; DIF: direct immunofluorescence; Ig: immunoglobulin.

**Table 6:** Suggested gene polymorphisms that are associated with LP.

Gene polymorphism of immune-related genes	
IFN gamma [147, 148]	
TNF [149, 150]	
TNF-*α* receptor 2 [151]	
IL-4 (only nonerosive OLP) [147]	
IL-6 [150]	
Haplotype IV of IL-10 [152]	
IL-18 [153]	
Tumor suppressor genes (e.g., P53) [154, 155]	
**HLA-A3** (In a Swedish population with LP [156] and two British populations with LP [157] and with familial LP [158])	
HLA-A5 (in a British population with LP [157])	
HLA-B7 (in a British population with familial LP [158], another study supported against [159])	
HLA-B8 (OLP) [2], a study supported against [159]	
HLA-B15 (in a Croatian population with OLP) [160]	
HLA-B18 inverse association with OLP in a Croatian population [160]	
HLA-Bw35 (CLP) [2]	
HLA-Bw57 (in a British population with OLP) [161]	
HLA-BW61 (in a Japanese population with OLP) [162]	
**HLA-DR1** (in wide range of ethnicities) [163–167], in a British population with CLP [167], in an Italian population with LP [168], and in an Arab population with CLP [166]	
**HLA-DRB1∗0101** (in a Sardinian population in Italy with CLP [169] and also with LP in a Mexican Mestizo population [169] and in a British population with CLP [167]),	
HLA-DR2 (in an Israeli-Jewish population with erosive OLP) [170],	
HLA-DR3 (in a Swedish population with erosive OLP) [171]	
HLA-DR9 (in Chinese population with erosive OLP) [172]	
HLA-DRW9 (in a Japanese population with OLP) [162]	
HLA-DR10 (in an Arab population with CLP) [166]	
**HLA-DQ1** (in a British population with CLP, [167] inverse association with OLP in a British population [161]	
**HLA-DQB1∗0201** (with vulvovaginal-gingival syndrome in two British populations) [81, 173]	
HLA-Te22 (in a Chinese population with erosive OLP) [172]; also it is highly associated with positive antinuclear antibodies in erosive OLP patients [174]	
rs2372736, defined at the chromosome 3p14-3q13 [175]	

Gene polymorphism of other genes	

Oxidative stress [176–179]	
PGE2 [180]	
Antithyroglobulin, antithyroid microsomal autoantibody, T3, T4 [181–183]	
Prothrombin [184]	
Epigenetic-associated genes (DNMT3B) [185]	
miRNA-146a [186] and miRNA-155 [186, 187]	
Nuclear factor-kappa B p65 [188]	

The ones in bold are considered more prevalent than the others.

**Table 7:** Clinical associations of LP.

Cutaneous diseases	
Chronic graft versus host disease [198–200]	
Alopecia areata [201, 202]	
Pemphigus vulgaris [203]	
Paraneoplastic pemphigus [204]	
Dermatitis herpetiformis [205, 206]	
Bullous pemphigoid [207–209]	
Atopic dermatitis [23]	
Psoriasis [210]	
Vitiligo [211–213]	
Morphea [214–217]	
Dermatomyositis [218]	
Lichen sclerosus et atrophicus [215, 219–221]	

Other diseases	

Liver disease [201, 222] (primary biliary cirrhosis, [223–225] primary sclerosing cholangitis [226])	
HCV infection [138, 142, 227]	
EBV infection [228–230]	
HPV infection [145, 230–232]	
HHV-7 [230, 233, 234]	
Ulcerative colitis [201, 202]	
Chronic gastritis, Helicobacter pylori [77, 235–237]	
Dyslipidemia [197, 238, 239]	
Anxiety [132, 135, 240–250] or stress [20, 251]	
Depression [132, 134, 135, 241, 242, 244–250, 252–255]	
Celiac disease [256–259]	
Myasthenia gravis and thymoma [260–266],	
Systemic lupus erythematosus [267–270]	
Sjögren's syndrome [271, 272]	
Multiple sclerosis [273]	
Hypothyroidism [274]	
Hashimoto thyroiditis	
Verruciform xanthoma [275]	

## Abbreviations

ABCG2: ATP-binding cassette transporter G2
CLP: Cutaneous lichen planus
DIF: Direct immunofluorescence
DLE: Discoid lupus erythematosus
FFA: Frontal fibrosing alopecia
HCV: Hepatitis C virus
HPV: Human papillomavirus
LP: Lichen planus
LPP: Lichen planopilaris
MF: Mycosis fungoides
NK cells: Natural killer cells
OLP: Oral lichen planus
SCC: Squamous cell carcinoma
Th: T helper
TLR: Toll-like receptor.
